# Enhancing laser speckle reduction by decreasing the pitch of a chiral nematic liquid crystal diffuser

**DOI:** 10.1038/s41598-021-83860-3

**Published:** 2021-03-01

**Authors:** David J. Hansford, Yihan Jin, Steve J. Elston, Stephen M. Morris

**Affiliations:** Department of Engineering Science, University of Oxford, Parks Road, Oxford, OX3 1PJ UK

**Keywords:** Engineering, Materials science, Optics and photonics

## Abstract

The artefact known as speckle can plague numerous imaging applications where the narrow linewidth of laser light is required, which includes laser projection and medical imaging. Here, we report on the use of thin-film chiral nematic liquid crystal (LC) devices that can be used to mitigate the influence of speckle when subjected to an applied electric field. Results are presented which show that the speckle contrast (a quantitative measure of the presence of speckle) can be significantly reduced by decreasing the pitch of the chiral nematic LC from 2700 to 244 nm. Further reduction in the speckle contrast can be observed by operating the diffuser technology at a temperature close to the chiral nematic to isotropic transition. At such temperatures, we observe a simultaneous improvement in the transmission of light through the device and a decrease in the electric field amplitude required for the minimum speckle contrast value. We conclude by presenting a laser projected image of the 1951 USAF target with and without the LC device to demonstrate the visual improvement as a result of the speckle reduction.

## Introduction

Speckle is a well-studied phenomenon that occurs when a coherent, or at least partially coherent, beam of light is scattered either by an optically rough reflecting surface or by travelling through a material with a random variation in its refractive index. Most surfaces can be considered as being optically rough, with high quality mirrors being a notable exception. Each point on a rough surface can be treated as a secondary source of light that contributes to the reflected light field. An observer with a finite aperture samples this optical field at a point in space and the intensity that is observed is then a summation of the complex optical fields (consisting of both amplitude and phase information) at that position. The variation in amplitude and phase that arises due to path differences between the observer and these ‘secondary emitters’ leads to a random amount of constructive and destructive interference, which in turn causes a random noise pattern across the observed light field. This granular intensity profile has been given the name ‘speckle’^1^.

Speckle can be observed in a wide range of applications either with (subjective) or without (objective) the use of image-forming optical configurations. Technological applications in which speckle can be observed includes laser projectors^2^ and optical coherence microscopy^3^ where, in both cases, it is considered an unwanted feature that should be removed. The degree of speckle present in an image with uniform average intensity can be quantified by the speckle contrast *C* of the interference pattern. This is found by dividing the standard deviation of the intensity values σ_*L*_ by the average intensity value *I̅*, and is effectively the reciprocal of the signal to noise ratio^1^,

$$C = \frac{{\sigma_{L} }}{I}$$

To reduce the speckle contrast, two or more statistically independent, and at least partially decorrelated, speckle patterns need to be superimposed. Under these conditions the random intensity fluctuations can be ‘averaged out’ across the image, depending upon the number of patterns used. Generally, speckle reduction techniques can be classified according to whether the statistically independent speckle patterns are created instantaneously or time sequentially. Time sequential methods take advantage of the finite integration time of the observer. For example, the human eye has an integration time of approximately 50 ms^4^. The techniques that can be employed to reduce the speckle contrast can also be further classified by the method by which these speckle patterns are mutually decorrelated.

Examples of four different approaches that have been successfully employed include spectral, spatial, angular and polarisation diversification. These different methods are not mutually exclusive and can be used in combination to further reduce speckle contrast: the total speckle reduction is typically the product of the speckle reduction of each method, assuming that each method produces speckle patterns that are statistically independent. One of the most common forms of speckle reduction involves passing a beam of coherent light through a rotating ground glass diffuser (RGGD). In this method, the diffuser creates a time-varying spatially-random phase perturbation across the beam leading to angular decorrelation that can reduce speckle contrast to values below *C* = 0.05^5–7^. Researchers have also considered combatting speckle noise by developing laser sources that have been engineered to have a low spatial coherence, and which have shown considerable promise when used to generate full-field images^8^. However, such an approach requires the use of very specific light sources (e.g. random lasers) and is not necessarily an optical component that could be retrofitted into existing imaging and projection display systems.

Alongside optical methodologies (i.e. ones that involve directly altering the coherence of the illumination source), there is also a growing body of research devoted to the development of numerical-based techniques that can emulate the process of speckle decorrelation. Such techniques achieve a reduction in the speckle contrast by computing a set of uncorrelated speckle patterns from a single recording, which when summed together follow the same dependence on the number of averaged independent speckle patterns, *N*, that is observed using optical techniques: namely, $1/\sqrt N$. For technologies such as digital holography, which is important for 3-dimensional coherent imaging, this alternative approach has proved to be particularly successful^9–12^. Nonetheless, optical-based techniques do have the advantage that no computational time is required to calculate a set of uncorrelated speckle patterns on the fly.

Liquid crystals (LCs) are a potentially useful material for speckle reduction as the optical properties can be altered externally with an electric field leading to phase, polarisation or intensity variation of the incident light that fluctuates in both time and space. Varying these properties as a function of time results in a time variation of the speckle spot intensities and positions, which leads to the creation of multiple, statistically independent speckle patterns that can then be summed together over a finite integration time so as to reduce the perceived appearance of speckle. This approach is equivalent to the time-averaging techniques that have been developed by research teams that have employed the use of colloidal-based material systems rather than those fabricated from liquid crystalline materials^13,14^.

To date, there have been numerous reports demonstrating speckle reduction using LC devices and materials. For example, previous studies have shown a reduction in the speckle contrast using: (1) a nematic LC with photo-isomerisable alignment layer that enables two orthogonal polarisation states to be created thereby leading to polarisation diversity^15^; (2) a chiral smectic ferroelectric LC (FLC) with an alternating field applied that creates a spatially and temporally random refractive index across the cell^16–18^; (3) surface and/or polymer stabilised FLC^19^ for polarisation diversity; (4) nematic LCs mixed with photocurable monomers for light scattering^20^, and (5) the use of a LC spatial light modulator (SLM) that applies multiple random phase masks corresponding to the Hadamard orthogonal function to create statistically independent speckle patterns at the observer^21^. These techniques, whilst they show promise, are not without their limitations such as a small amount of speckle reduction, complex electric field profiles, and/or the use of expensive and bulky components (such as an SLM).

In a previous study, we showed that a positive dielectric anisotropy chiral nematic LC with a pitch of 250 nm and doped with an ionic dopant (cetyltrimethylammonium bromide—CTAB ) can cause a spatially and temporally random phase perturbation to incident laser light when operated in a dynamic scattering mode by subjecting the LC to a low frequency (< 100 Hz) square wave electric field of sufficiently large amplitude^22^. The random perturbation to the phase of light can, in turn, result in a reduction in the observed speckle contrast. The purpose of this paper is to demonstrate that speckle reduction can be observed in chiral nematic LC mixtures without the need for an ionic dopant such as CTAB and to show that the speckle contrast can be reduced to lower values than those reported previously in^22^. Furthermore, we consider how the pitch of the chiral nematic LC helix influences the magnitude of the speckle reduction as well as the electric field amplitude and applied frequency required to achieve optimum speckle reduction. The results show that a reduction in the pitch, *p*, from 2700 to 244 nm leads to a reduction in the speckle contrast. However, the transmission of the laser light through the device reaches a minimum for pitch values of *p* = 0.5–1 μm before increasing with a further reduction in the pitch. The results indicate that there is an inverse relationship between the pitch and both the electric field amplitude and frequency required for peak device performance. Finally, we consider how changes in the temperature can further reduce the speckle contrast and concluding by presenting static images recorded for a monochromatic laser imaging system that demonstrates the noticeable improvement in the projected images when using these LC devices. Importantly, no loss in the resolution of the optical imaging system is observed.

## Results and discussion

### Reducing laser speckle using the dynamic scattering mode

**Figure 1:**
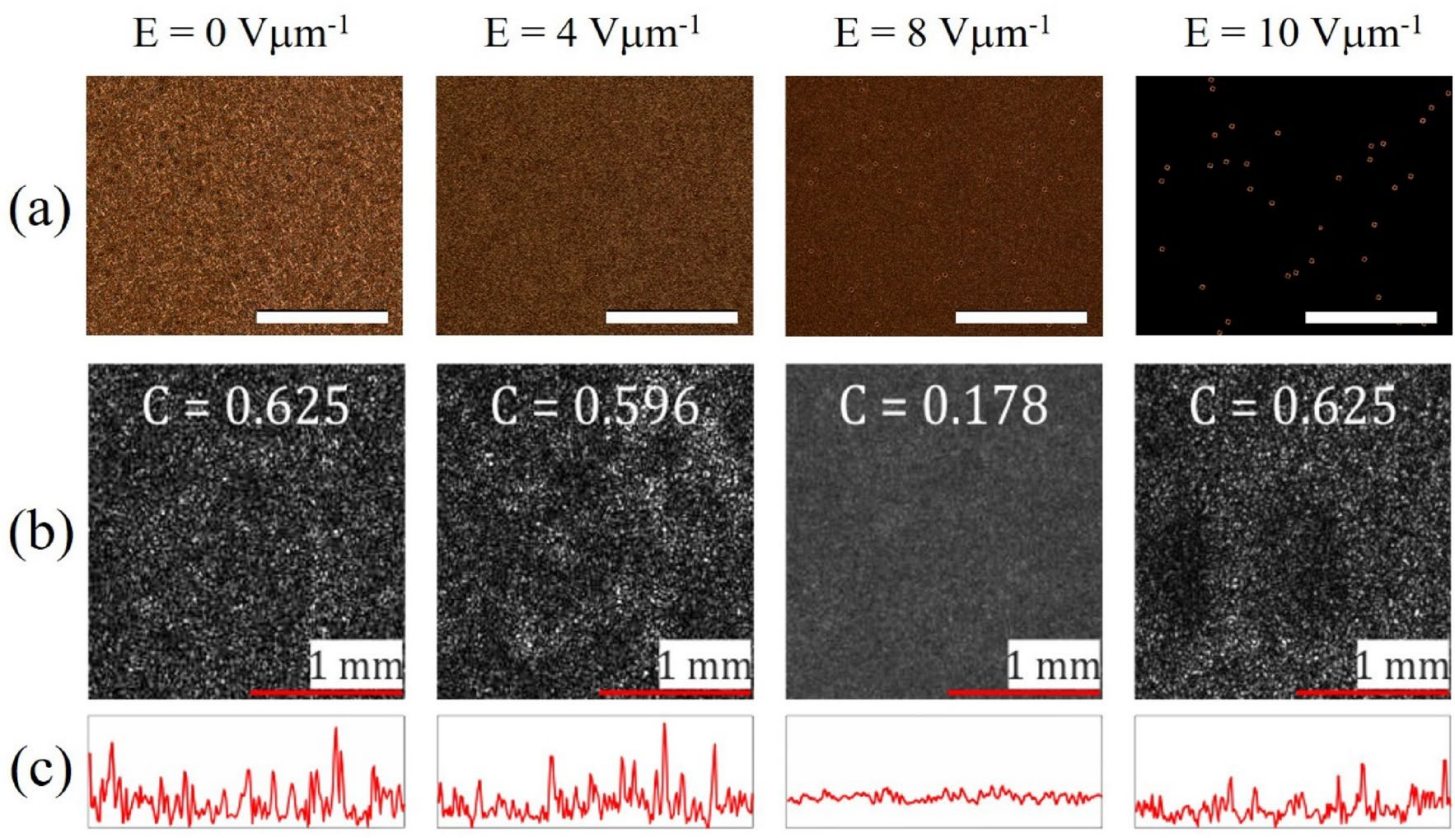
Speckle reduction characteristics of a chiral nematic LC device (E7 + 2.5 wt% BDH1281, pitch 517 nm) when subjected to different amplitudes of a square-wave electric field at a frequency of 40 Hz (**a**) Optical polarising microscope images of the LC device (the 20 μm-diameter spacer beads can be seen in all images, although they are most obvious for the image recorded at E = 10 Vµm^−1^). Scale bars are 500 µm. (**b**) Corresponding images of the speckle pattern formed on a white screen after the beam from a He–Ne laser has passed through the LC device. The same electric field is applied as those used in (**a**). Images were captured by the CCD camera with a 50 ms exposure time and normalized by the average intensity. (**c**) Plots of the intensity distribution of the central line of pixels across the width of each image of the speckle pattern presented in (**b**). All measurements were carried out at 25 °C using a 20 µm-thick device.

An example of how the speckle pattern and the corresponding speckle contrast changes with the use of a chiral nematic LC device that is subjected to different electric field amplitudes is presented in Fig. 1. In this case the chiral nematic LC mixture consists of the nematic host (E7, Synthon Chemicals Ltd.) and 2.5 wt% of the high twisting chiral dopant, BDH1281 (Merck). This mixture was found to have a pitch of *p* = 517 nm at *T* = 25 °C. For this mixture, the critical electric field, *E*_c_, required for the chiral nematic-nematic transition was found to be *E*_c_ = 10 Vµm^−1^. Polarising microscope images are shown for four different electric field amplitudes, *E*, at the same applied frequency (*f* = 40 Hz) (Fig. 1a). The first image shows a static focal conic state at *E* = 0 Vµm^−1^ which then transforms to a turbulent, dynamic scattering state with the application of an electric field (examples are shown for amplitudes of *E* = 4 and 8 Vµm^−1^). The final image shown (far right) is for a homeotropically-aligned nematic state at 10 Vµm^−1^, where the helical structure has been unwound by the large field amplitude. The image appears dark, in accordance with a nematic LC aligned in the homeotropic state when viewed between crossed polarisers, except for the bright circular regions which correspond to the distortion in the director field around the spacer beads.

Corresponding images of the speckle pattern recorded for these electric field conditions are presented in Fig. 1b, with the calculated speckle contrast, *C*, appearing in the inset of each figure. It can be seen that for the static focal conic state and the homeotropic nematic state, the value for the speckle contrast is the same as that recorded for the laser without the LC device (i.e. *C* = 0.625). For both of these field conditions, the granular intensity pattern is very noticeable. However, for electric field amplitudes that correspond to the turbulent dynamic state, the speckle contrast reduces first to *C* = 0.596 at *E* = 4 Vµm^−1^ before decreasing further to *C* = 0.178 when the amplitude is increased to *E* = 8 Vµm^−1^. Line profiles of the intensity can be found in Fig. 1c for each speckle pattern, where it can be seen that the variation in the intensity flattens out for *E* = 8 Vµm^−1^ as expected from the smaller value of the speckle contrast and the more uniform intensity profile seen in the image presented in Fig. 1b.

**Figure 2:**
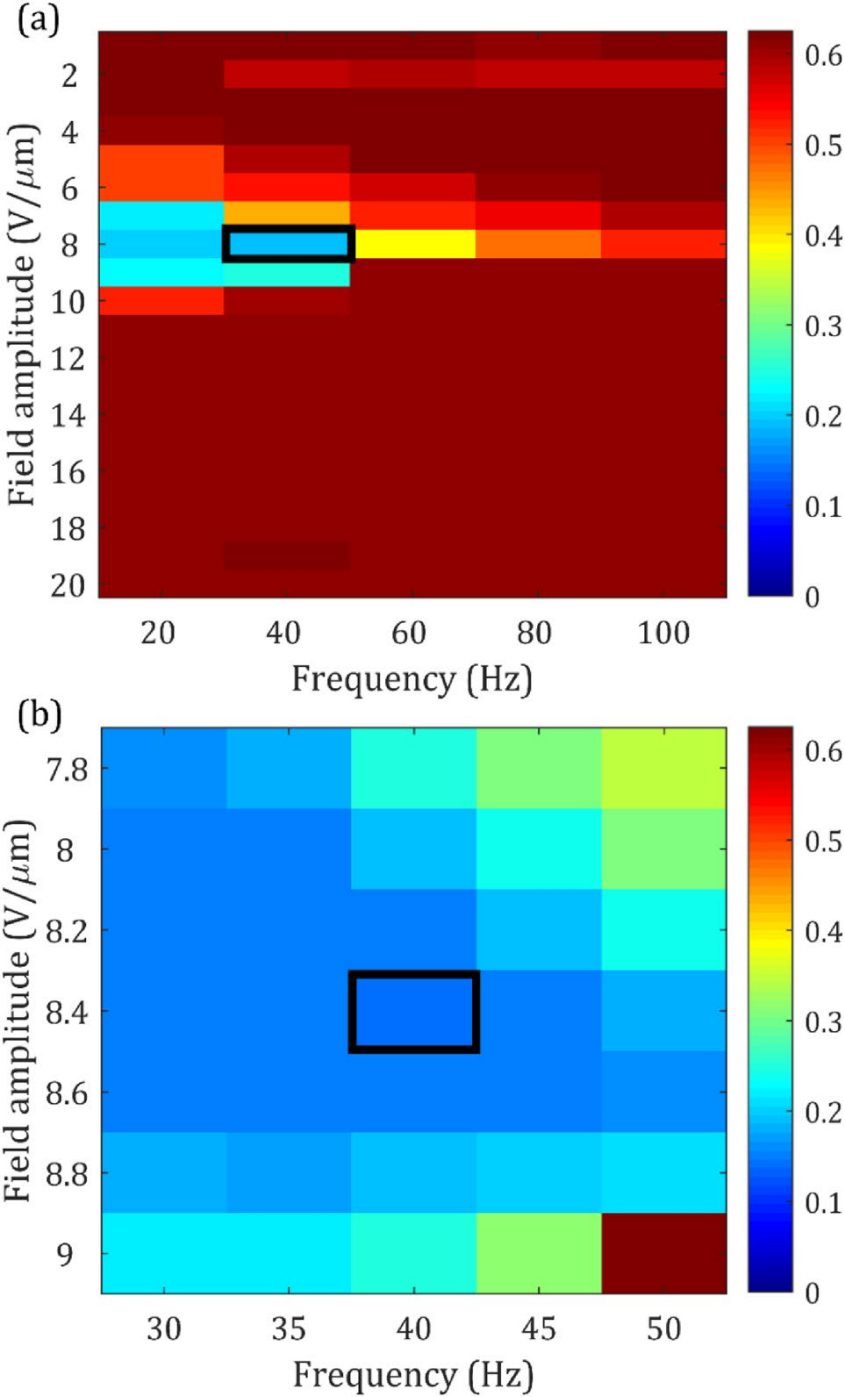
Colour maps showing the variation in the Speckle Contrast, *C* (legend shown on the secondary axis) for different amplitudes and frequencies of a square wave electric field applied to the chiral nematic LC device (E7 + 2.5 wt% BDH1281, pitch 517 nm). (**a**) Electric field and frequency increments of 2 Vµm^−1^ and 20 Hz, respectively. (**b**) higher resolution measurements–electric field and frequency increments of 0.2 Vµm^−1^ and 5 Hz, respectively. The black boxes in (**a**) and (**b**) represent the electric fields corresponding to the smallest speckle contrast ratio, *C* (maximum speckle reduction). All measurements were carried out at 25 °C using a 20 µm-thick device.

In accordance with our previous study for ionic mixtures^22^, we find that the speckle contrast reduces with increasing field amplitude up until the helical structure unwinds, at which point all dynamic scattering ceases. To identify the electric field conditions (amplitude and frequency) for which the speckle contrast is a minimum, we carry out a series of measurements across electric field amplitudes ranging from *E* = 0 Vµm^−1^ to *E* = 20 Vµm^−1^ and from frequencies ranging from 0 to 100 Hz. The variation in the speckle contrast with field conditions is shown in Fig. 2 in the form of a colourmap, where the legend on the righthand side depicts the value of the speckle contrast, *C*. Red sections in the map represent little-to-no speckle reduction (speckle contrast is virtually unchanged i.e. *C *≈ 0.625) while dark blue regions represent high-levels of speckle reduction (speckle contrast, *C* < 0.2). From the map in Fig. 2a, which shows the results for a sweep of the field conditions in 2 Vµm^−1^ and 20 Hz increments, it is clear that the minimum in the speckle contrast (peak speckle reduction) occurs at an electric field amplitude of *E* = 8 Vµm^−1^ and a frequency of *f* = 40 Hz (highlighted by the black box). At this amplitude and frequency, we find that the speckle contrast has reduced from *C* = 0.625 to a speckle contrast of *C* = 0.19.

Having established the approximate amplitude and frequency for minimum speckle contrast, the next step was to study the cell at a higher resolution of electric field conditions close to the region where the peak speckle reduction was observed. Towards this end, the electric field amplitude was varied from 7.8 to 9.0 Vµm^−1^ in increments of 0.2 Vµm^−1^, and the frequency was varied from 30 to 50 Hz in increments of 5 Hz. The resulting colourmap is shown in Fig. 2b. With this finer resolution, the minimum speckle contrast was now found to occur more precisely at *E* = 8.4 Vµm^−1^ and *f* = 40 Hz where the speckle contrast was *C* ≈ 0.15.

### The importance of the pitch

**Figure 3:**
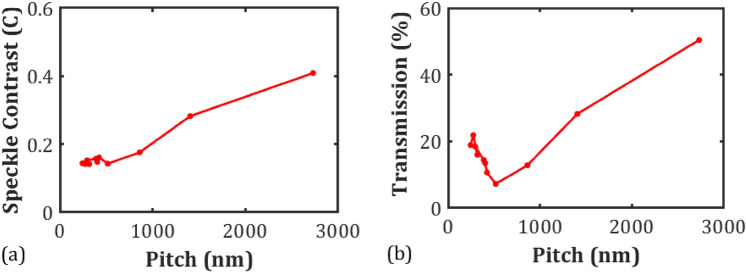
Plots of the minimum speckle contrast (**a**) and transmission (**b**) as a function of the pitch of the chiral nematic LC device. Measurements were carried out at 25 °C using a 20 µm-thick device for mixtures consisting of E7 and the chiral dopant BDH1281 (0.5–6.4 wt%). The solid red lines represent a linear interpolation to guide the eye.

We now consider whether the magnitude of the helical pitch has any impact on the minimum speckle contrast that can be achieved. For this study, a range of pitch values are presented for mixtures consisting of the nematic host E7 and different concentrations by weight of the chiral dopant, BDH1281. The influence of a change in pitch on the electric field amplitude and frequency required for maximum speckle reduction is explored and for each pitch we determine the minimum speckle contrast value that can be achieved. Figure 3 shows the average speckle contrast measured during a 5-min steady state test at the electric field amplitude and frequency for which the lowest value of the speckle contrast was observed. The data show that for the chiral nematic samples with a pitch value below 500 nm the speckle contrast is relatively independent of the pitch and is approximately constant at a value of *C* ≈ 0.15. However, as the pitch is increased above this value the speckle contrast begins to increase with the pitch, rising to a speckle contrast of *C* ≈ 0.4 for *p* = 2700 nm, which represents only a 36% reduction in the speckle. Evidently, the data demonstrates that by reducing the pitch of the chiral nematic LC from 2700 to 244 nm the speckle contrast (speckle reduction) can be decreased (increased).

For electrohydrodynamic instabilities (EHDI) in chiral nematic LCs the size of the domains that exist at lower electric field amplitudes has been shown to be of the order of the pitch^23^. Therefore, one might expect that a mixture with a shorter pitch would exhibit more spatial variation of the birefringence across layer, which would lead to increased scattering. Also, mixtures with long pitches (> 4 μm) typically exhibit an electric field-induced fingerprint texture rather than the focal conic state^24,25^, which will result in less scattering. This is consistent with the limited speckle reduction achieved for the long pitch mixtures considered in this study.

In Fig. 3b, the transmission, which is defined as the ratio of light intensity at the projection screen to that measured without a LC device in place, is presented as a function of the pitch. The values shown are the average transmission measured over the 5-min steady state test at the electric field conditions for which a minimum in the speckle contrast was observed. From this plot it can be seen that as the pitch decreases from a value of *p* = 2700 nm, the transmission reduces with the pitch, reaching a minimum around *p* = 500 nm. However, a further shortening of the pitch results in an increase in the transmission. It is worth noting that the pitch corresponding to the minimum transmission does not coincide with an overlap of the band-gap with the wavelength of the laser source. Specifically, the mixture for which the minimum transmission was observed was the mixture, E7 + 2.5 wt% BDH1281, which was found to exhibit a band-gap from 785 to 902 nm. This is at longer wavelengths than the He–Ne laser used for the measurements of the speckle contrast (*λ* = 632.8 nm).

The same reasoning applied to the speckle contrast could also be applied to the transmission behaviour observed for longer pitch mixtures. However, the existence of a transmission minima is more difficult to explain. When considering using a LC cell for speckle reduction in imaging, maximum transmission would be highly desirable while retaining minimum speckle contrast. As a result, the choice of the pitch value is rather important as it is desirable to obtain the smallest possible speckle contrast value whilst maximising the transmission (Fig. 3b). Therefore, chiral dopants with a high helical twisting power should be considered for future studies.

**Figure 4:**
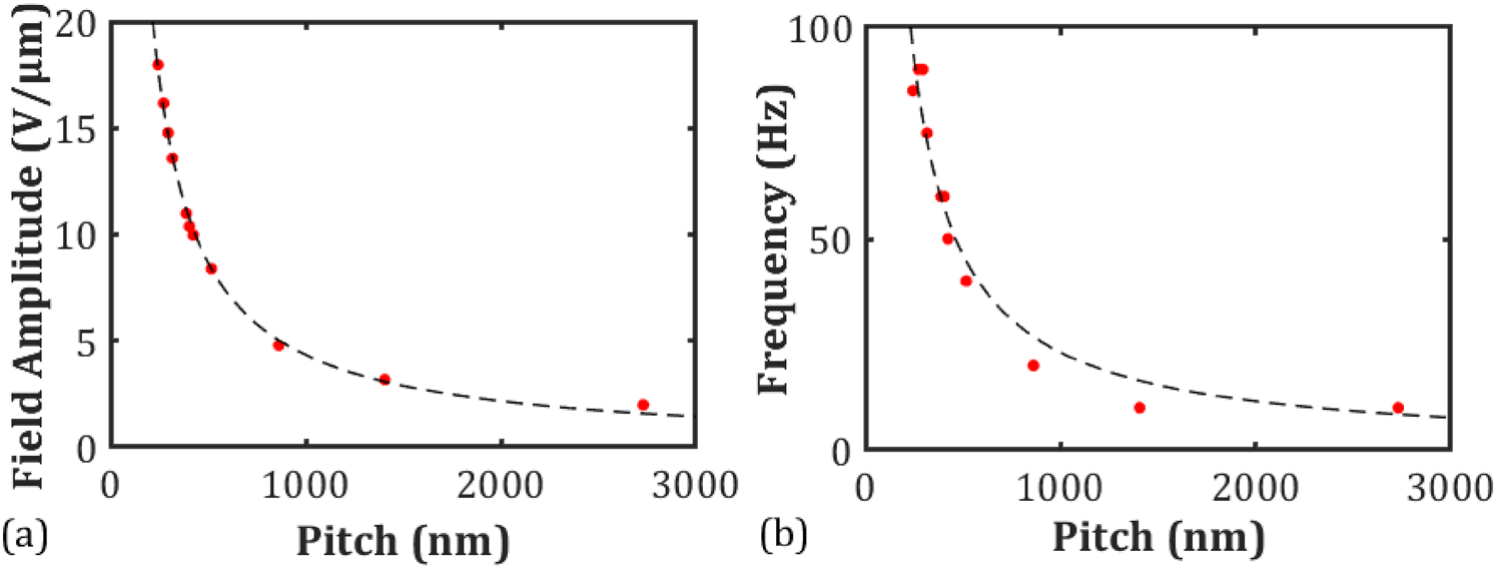
Plot of the amplitude (**a**) and square-wave frequency (**b**) of the applied electric field at the minimum speckle contrast (maximum speckle reduction) as a function of the pitch. Each cell was 20 μm-thick and the cell temperature throughout the measurements was held at *T* = 25 °C. Data points represent the measured values and the dashed black lines are fits of the form *ax*^−1^.

Results for the dependence of the electric field amplitude corresponding to the minimum speckle contrast as a function of the pitch are presented in Fig. 4a. Values for the electric field amplitude and frequency were obtained from the high-resolution scans in accordance with the process demonstrated in Fig. 3b. The results demonstrate that there is an inverse relationship between the pitch of the chiral nematic LC and the electric field amplitude required for the minimum speckle contrast (maximum speckle reduction). At the longest pitch value tested (*p* = 2700 nm), *E* ≈ 2 Vµm^−1^, which increases to *E* = 20 Vµm^−1^ for the shortest pitch considered in this work. Any decrease in the pitch results in an increase in the electric field amplitude as $E \propto 1/p$.

Theoretical studies of EHDI distortions in chiral nematic LCs that are subjected to ac electric fields applied parallel to the helical axis were carried out many years ago by Helfrich and separately by Hurault assuming that the cell gap is significantly larger than the helical pitch^26,27^. Subsequent experimental work by Rondelez and co-workers supported the results from the theoretical studies^28^. In these collective works, it was predicted that there is an inverse relationship between the threshold voltage for the onset of EHDI and the square root of the pitch. However, we find that the field required to achieve minimum speckle contrast is considerably larger than the threshold field for EHDI. Speckle contrast maps for each mixture indicate that the field required for the minimum speckle contrast was always just below the threshold field needed for the chiral nematic-nematic phase transition. The relationship between the threshold field required to unwind the helix and the pitch is *E* ∝ *p*^−1^, which is the same as the relationship shown in Fig. 4a.

The dependence of the frequency that corresponds to the lowest value of the speckle contrast is presented as a function of the pitch of the chiral nematic LC in Fig. 4b. It is shown that the frequency for maximum speckle reduction is also approximately inversely proportional to the pitch. Frequencies of the applied field below 10 Hz were not tested because at such low frequencies, DC effects from carrier injection become significant.

In the work of Hurault a relaxation frequency, *ω*, for positive dielectric anisotropy chiral nematic LCs is provided, which follows the form^27^

1 $$\omega = \left( {\frac{\pi }{d}} \right)\left( {\frac{2\pi }{p}} \right)\left( {\frac{{\overline{K}}}{\gamma }} \right)$$

where $\overline{K}$ is the average value of the Frank elastic constants, *d* is the cell thickness, *p* is the pitch and γ is the rotational viscosity coefficient. Using typical values for E7 of $\overline{K}$ = 14 pN and γ = 0.15 Pa s along with *d* = 20 μm and *p* = 0.5 μm, a relaxation frequency of *f*≈50 Hz is obtained. This value is remarkably close to the frequency recorded for the minimum speckle contrasts of the chiral nematic LC mixture consisting of E7 and BDH1281 with a pitch of *p* = 500 nm, which was found to be *f* = 40 Hz. Furthermore, Eq. (1) follows the same inverse relationship between frequency and pitch as observed in Fig. 4b.

The sample that was found to give the largest reduction in the speckle contrast in this study was the chiral nematic LC doped with 4.7 wt% of the high twisting power chiral dopant BDH1281, which had a pitch of *p* = 317 nm at *T* = 25 °C. Specifically, this mixture was found to be capable of reducing the speckle contrast to *C* = 0.141 at *E* = 13.6 Vµm^−1^ and *f* = 75 Hz: a reduction of 77% from *C* = 0.625. The corresponding transmission through the device under these field conditions was found to be 16.0%. However, by increasing the concentration of chiral dopant further, leading to a shorter pitch (5.1 wt%, *p* = 273 nm), it was found that the speckle contrast reduced to almost the same level (*C* = 0.142) but with an improved transmission of 21.9%.

### Camera integration time

**Figure 5:**
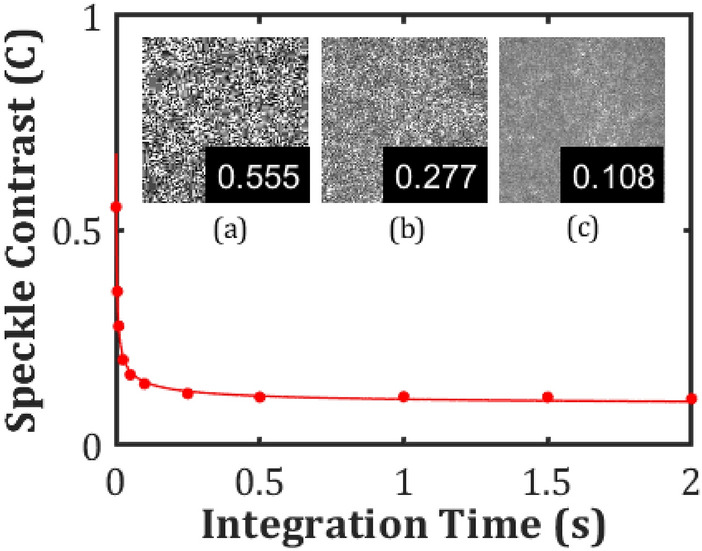
Speckle contrast measured after passing through a chiral nematic LC device (E7 + 4.7 wt% BDH1281, pitch 310 nm) under a square wave electric field of amplitude 13.6 Vµm^−1^ and a frequency of 75 Hz at a range of camera integration times. The cell thickness was d = 20 µm, cell temperature T = 25 °C. The data points were obtained from measurements and the solid red line is a fit of the form *ax*^−0.5^ + *b* which shows that *C* reduces approximately by $1/\sqrt \tau$. Inset speckle images shown for (**a**) 1 ms, *C* = 0.555, (**b**) 10 ms, *C* = 0.277, (**c**) 2 s, *C* = 0.108 integration time and speckle contrast values, respectively.

The underlying principle governing the use of the LC device is that the speckle contrast can be reduced if a number of speckle patterns are superimposed upon each other within the integration time of the detector, in this case the CCD camera. As already stated, passing the coherent laser beam through a LC cell undergoing EHDI has the effect of applying a time-varying spatially random phase perturbation on the incoming wavefront. Goodman^1^ showed that for *N* statistically independent speckle patterns superimposed during one integration period, the speckle contrast is reduced by a factor of $1/\sqrt N$. For our device, this would correspond to a relationship of the form of $1/\sqrt \tau$, where $\tau$ is the integration time of the detector. This is because there is a direct relationship between the integration time and the number of statistically independent speckle patterns observed by the camera as a result of LC director fluctuations in the device; the longer the time the more patterns that are captured. The relationship between the speckle contrast and the integration time of the detector can be observed clearly from our experimental data presented in Fig. 5. The cell was maintained under constant electric field conditions for each measurement and the observed speckle contrast is seen to reduce as the integration time of the CCD camera increases following a $1/\sqrt \tau$ dependency.

Examples of the speckle contrast maps for four different camera exposure times can be seen in Figure S1 in the Supplementary Information. The results show that the electric field amplitudes and frequency over which the speckle contrast significantly decreases narrows with a reduction in the camera exposure time. In accordance with Fig. 5, the minimum value of the speckle contrast is also seen to increase with a decrease in the exposure time. For our study, we are primarily concerned with an experimental configuration that mimics the response of the human eye and therefore a fixed integration time of 50 ms has been selected for our measurements. However, if the integration time is not a fixed quantity, then it is important to note that the minimum speckle contrast that can be achieved for a specific LC device operated at a single temperature depends upon three factors: the electric field amplitude, frequency of the applied field and the camera/detector integration time.

### Temperature

**Figure 6:**
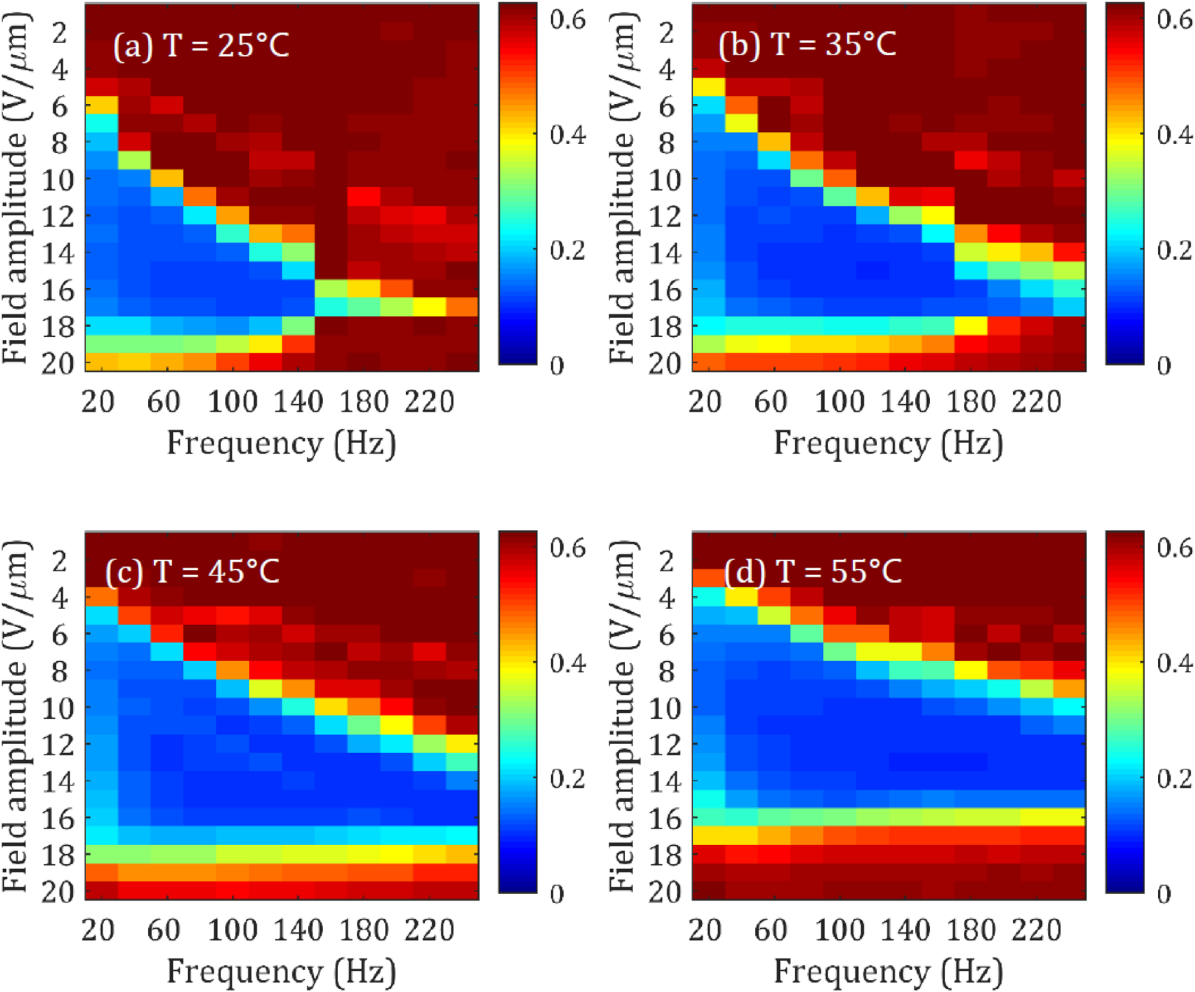
Speckle contrast maps measured for the chiral nematic LC device (E7 + 5.1 wt% BDH1281, pitch 273 nm) for four different operating temperatures. The cells tested were nominally 20 µm-thick and the cell temperature throughout measurements was (**a**) T = 25 °C, (**b**) T = 35 °C, (**c**) T = 45 °C, (**d**) T = 55 °C.

Thus far, it has been demonstrated that the magnitude of the pitch can significantly influence the minimum speckle contrast value that can be achieved using the LC device. We now consider the influence that the environmental temperature has on the amount of speckle reduction. Colour maps for four different temperatures are presented in Fig. 6, ranging from T = 25 °C to T = 55 °C (just below the clearing temperature of the chiral nematic LC mixture). As the temperature is increased from 25 to 55 °C we see that the range of field amplitudes and frequencies for which a significant speckle reduction is observed (indicated by the blue region in the figure) is increased. For example, at a temperature of T = 25 °C we see that the lowest values for the speckle contrast occur at relatively low frequencies and relatively large electric field amplitudes. To illustrate this point, if we consider a frequency of *f* = 100 Hz we see that amplitudes of around *E* = 14–18 Vµm^−1^ are required to reduce the speckle contrast to *C* < 0.2. However, by increasing to T = 55 °C we see that at the same frequency (*f* = 100 Hz), the field amplitude required to reduce the speckle contrast to values of *C* < 0.2 has dropped to *E* = 8–15 Vµm^−1^.

**Figure 7:**
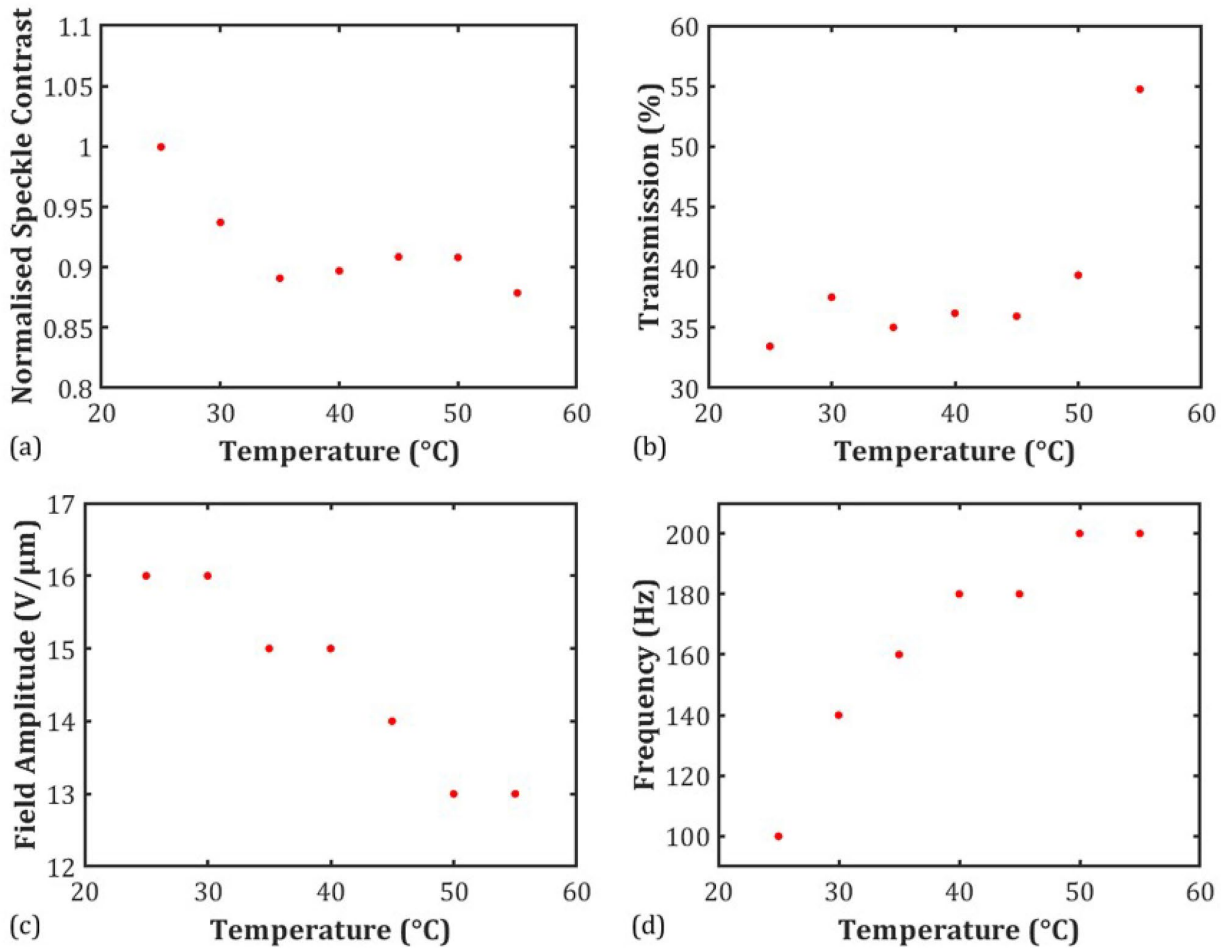
The temperature dependence of (**a**) minimum speckle contrast normalised to the value of *C* at 25 °C, (**b**) transmission, (**c**) electric field amplitude and (**d**) frequency at the minimum speckle contrast conditions. Devices were nominally 20 µm-thick and contained the chiral nematic LC mixture, E7 + 5.1 wt% BDH1281, which had a pitch of *p* = 273 nm at 25 °C.

In terms of the variation in the minimum speckle contrast value, the normalised speckle contrast (normalised to the value at T = 25 °C) is plotted as a function of the temperature in Fig. 7a. In this case it can be seen that *C* reduces in magnitude by almost 15% with an increase in temperature. It is well-known that the birefringence reduces with increased temperature^29^ and, as a rule of thumb, a decrease in the birefringence leads to a decrease in the speckle reduction as a result of reduced light scattering. However, the viscosity also decreases with an increase in temperature^30^, which enables the local director to reorient more freely during the EHDI process thus increasing the rate of fluctuations across the device. This effect is analogous to increasing the exposure time of the camera, as shown in Fig. 5, as a decrease in the viscosity would serve to increase the number of independent speckle patterns generated per unit time, which would reduce the magnitude of the speckle contrast.

Encouragingly, alongside a decrease in the speckle contrast we also observe an increase in the transmission (Fig. 7b), indicating greater light throughput, a decrease in the required electric field amplitude from 16 to 13 Vµm^−1^ (Fig. 7c) and an increase in the drive frequency from 100 to 200 Hz (Fig. 7d). All of these changes are favourable in terms of device performance: in the latter case the increase to a higher value of the drive frequency ensures that the device can be operated at electric field conditions away from the low frequencies where the unwanted build-up of ionic species might occur.

### Projected image

**Figure 8:**
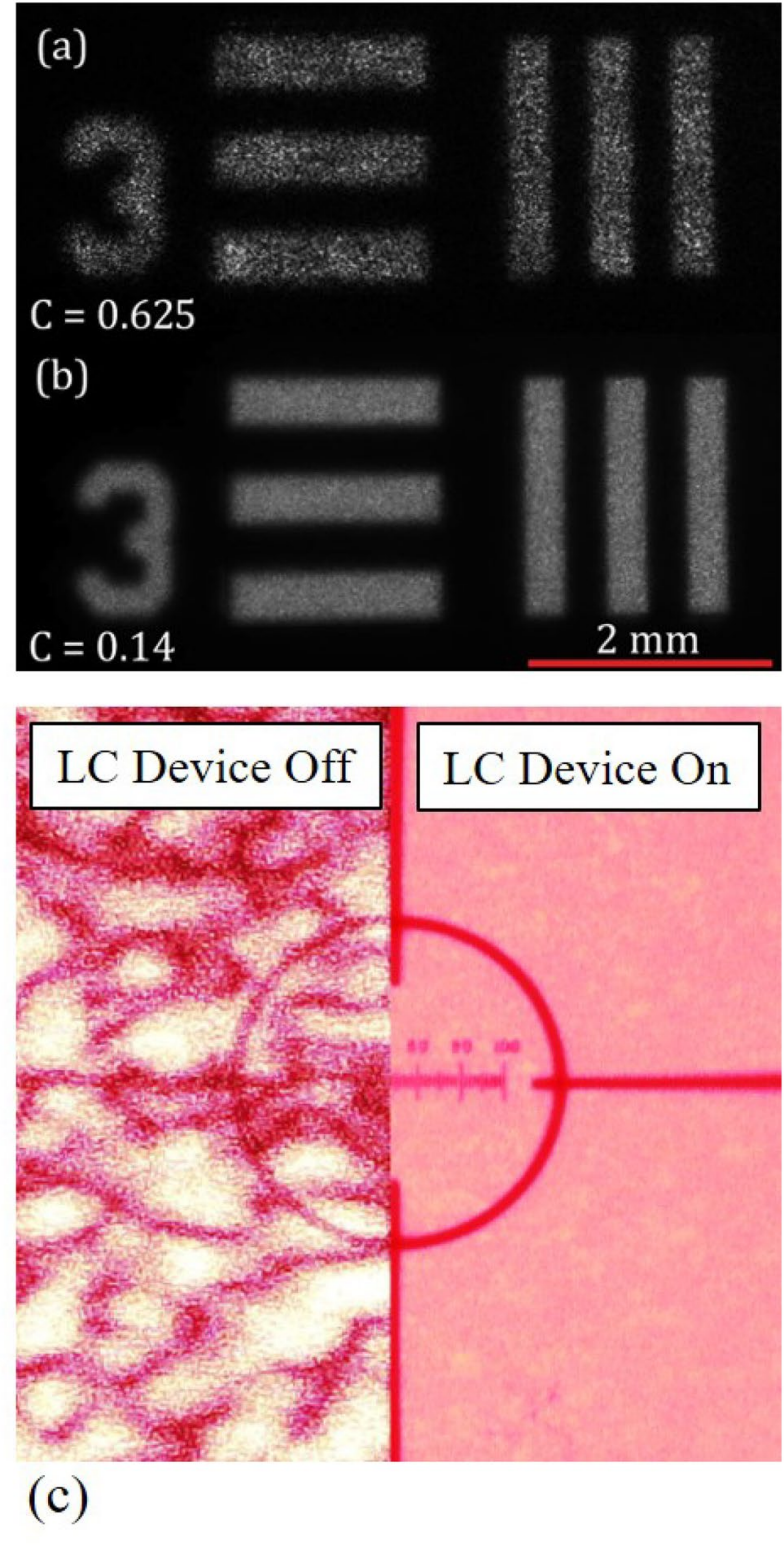
Images captured by the CCD of the white screen when an image of the 1951 USAF target is generated without (**a**) and with (**b**) a chiral nematic LC device (E7 + 4.7 wt% BDH1281, pitch = 310 nm). (**c**) Image showing that the resolution is preserved during the speckle reduction process. The left-hand and right-hand images show the speckle pattern for the case when the LC speckle reducer is switched off or on, respectively. An image of a microscope stage micrometer can be seen showing a scale bar from 0 to 100 µm. The device thickness was nominally 20 µm and measurements were carried out at T = 25 °C.

To illustrate, visually, the speckle reduction improvement afforded with the LC device used in this study, photographs of laser projected images are shown in Fig. 8. For this experiment, the He–Ne laser is propagated through the 1951 USAF target and the resultant image is projected onto a white screen using the set-up presented in Figure S1. Images were recorded both with and without the LC device in the path of the laser beam. In the upper panel (Fig. 8a), a section of the USAF target is shown without the LC device in the path of the laser. Here it can be seen that while the horizontal and vertical bars and the number 3 can be identified, the intensity is extremely nonuniform within the borders of these features due to the presence of speckle, which clearly deteriorates the quality and visual appeal of the image. From our measurements, we find that the speckle contrast in this image has a value of *C* = 0.625, which is consistent with the $1/\sqrt 2$ expected for this experimental configuration.

With the insertion of the LC device (Fig. 8b), we see that the features are noticeably improved with the addition of the LC device operating at the electric field conditions for which maximum speckle reduction is observed (i.e. a speckle contrast of C = 0.14). The 78% reduction in the speckle contrast clearly has an observable effect on the quality of the image that is produced. Further improvement is expected through material/mixture optimisation and it anticipated that such devices could lead to speckle contrast values below *C* = 0.1, thus approaching the limit beyond which speckle can no longer be perceived (*C* = 0.04).

Maintaining the resolution of an imaging or display system whilst reducing the speckle noise is of paramount importance for any speckle reducing technology. To verify that there is no loss of resolution when our LC speckle reducer is employed, we have recorded images of the speckle pattern when a microscope stage micrometer is imaged onto the screen. The experimental results are presented in Fig. 8c where it can be seen that the resolution of the smallest features on the micrometer are clearly discernible when the LC diffuser is switched on and that the ability of the optical system to resolve the lines on the micrometer scale is unaffected by the LC device. In contrast, the lines and features on the micrometer slide cannot be seen clearly when the LC device switched off due to the noise introduced by the speckle pattern.

We conclude this study by considering how the performance of the LC speckle reducer presented here compares with other optical diversification techniques. In this work, we have shown that, under the right experimental conditions, the speckle contrast can be reduced from *C* = 0.625 to *C* = 0.14 (a 78% reduction in speckle). This reduction compares well with results reported for other optical time-varying techniques such as those applied to digital holography as well as those involving the use of a spatial light modulator, where an 80% reduction was observed^31–33^. Our results also compare very favourably with previous work involving liquid crystalline materials where speckle reduction is typically of the order of 50%^18^. It is important to note that there is still further scope, through material optimisation, to reduce the speckle contrast further. For example, through the use of additional scatterers or by decreasing the response time of the LC it may be possible obtain speckle contrast values below *C* = 0.1.

## Conclusions

In summary, we have shown that by varying the pitch of a chiral nematic LC (based upon the composition of E7 + chiral dopant) the speckle contrast (when measured using a camera integration time of 50 ms) is reduced from *C* ≈ 0.4 for *p* = 2700 nm to *C* ≈ 0.15 for *p* = 270 nm. A further reduction in the minimum speckle contrast value can be obtained by increasing the device temperature to close to the clearing temperature of the mixture. By combining short pitch chiral nematic LC devices with operating temperatures approaching the clearing temperature, it is possible reduce the speckle contrast by more than 80% from the value of *C* = 0.625 recorded when no LC device was used, i.e. the value obtained corresponding to just the illumination of the He–Ne laser on the white screen. Configuring the LC device as outlined in this study enables speckle contrast values of C ≈ 0.1 to be reached, which is approaching the limit for speckle not to be observed. It is also found that operating at higher temperatures leads to the complementary benefits of a 36% increase in the light transmission through the device and a 20% decrease in the electric field required to generate the minimum speckle contrast. The results in this study provide an important guide as to the design and development of thin-film speckle reducers based upon LCs for deployment in laser projection and imaging systems.

## Methods

### Materials

The mixture components used in this work were weighed using a precision micro-balance (Mettler Toledo AB104-S) with an accuracy of ± 0.05 mg. For this work the well-characterised eutectic mixture, E7 (Synthon Ltd), was chosen as the nematic host as it is liquid crystalline at room temperature and its macroscopic physical properties such as the refractive indices, dielectric permittivities and elastic constants have been measured using a variety of techniques. To form a chiral nematic LC phase, the nematic host was dispersed with a low concentration by weight of the high twisting power chiral dopant BDH1281 (Merck), which had a helical twisting power of *β* = 72 µm^−1^ in E7. For this work a number of mixtures were prepared to study the influence of the pitch with concentrations ranging from 0.5 to 6.4 wt% of the chiral dopant. Each mixture was then heated to ∼ 10 °C above the clearing temperature and held at this temperature for at least 12 h to achieve complete thermally assisted diffusive mixing of the components. The resultant mixtures were found to exhibit a right-handed helix with pitch values ranging from *p* = 244 to *p* = 2700 nm. For the mixtures that exhibited a reflection band within the 350–1100 nm spectral range at a temperature of *T* = 25 °C (determined using an ultraviolet–visible spectrometer, Agilent Cary 8454 UV–Vis), the pitch was calculated from a combination of the birefringence (*Δn*) of E7 and the width of the photonic band gap, *Δλ*, according to the relationship *p* = *Δλ*/*Δn*. For mixtures that had a photonic band gap outside of the spectral range of the UV–Vis spectrometer (i.e. at longer wavelengths), the pitch was determined by extrapolating a linear plot of the inverse pitch (*p*^−1^) as a function of the concentration by weight (*c*_*w*_) (see Figure S2 in the Supplementary Information) as the two parameters are related through the expression *p*^−1^ = (β*c*_*w*_*e*_*e*_) where *β* is the helical twisting power and *e*_*e*_ is the enantiomeric purity, both of which remain constant for the same combination of nematic host and chiral dopant. In contrast to our previous study^23^, we found that the mixtures consisting of E7 + BDH1281 exhibit dynamic scattering without the need for additional ionic doping such as CTAB.

After thermal mixing, the LC mixture was then filled into thin glass cells using capillary action. The glass cells used in this work were commercially-available INSTEC LC2 cells that have a nominal cell gap of *d* = 20.0 μm and consisted of a transparent electrode coating of Indium Tin Oxide (ITO) (thickness 23 nm) that was patterned onto the inner surfaces of the glass substrates creating a 5 mm × 5 mm active region at the centre of the cell. On top of each ITO layer, a rubbed polyimide alignment layer was deposited onto the inner surface of each substrate. The rubbing directions were oriented such that they were aligned antiparallel to one another. The thickness of each glass cell was determined by the spacer beads that were spray-coated across the entire glass substrate including the active electrode areas. The actual thickness was determined using an interference method with white light illumination at normal incidence. It should be noted that for future work it would be more desirable for the spacer beads to be confined to regions outside of the active area so as to avoid any unwanted increase in the speckle. Examination of the devices on a polarising optical microscope (Olympus BX51-P) revealed that the components had been uniformly dispersed.

### Speckle characterisation

The experimental setup used to measure the Speckle Contrast is shown in Figure S3. Briefly, a single mode, continuous wave, linearly polarised Helium–Neon laser (JDS Uniphase 1122P, λ = 632.8 nm) was used as the coherent light source to illuminate the LC device. This device was placed on a hot-stage connected to a controller (INSTEC mK1000) to ensure that it was maintained at a constant temperature throughout each experiment. Temperatures used in the study of the speckle contrast vary from 25 to 55 °C. The controller has a temperature resolution of 0.001 °C.

An alternating electric field was generated inside the LC device using the dual-channel function generator (Tektronix AFG 3022) and voltage amplifier (FLC Electronics F10AD) described previously. A 10 × microscope objective (Olympus UPlanFL N, NA = 0.3), placed directly after the LC device, was used to limit the divergence of the scattered beam transmitted through the LC cell before an absorptive neutral density filter (NDF) (Thorlabs NEK01) reduced the intensity of the beam observed at the camera (CCD) to ensure that the sensor was not over-exposed. At the same time, it was important to retain the maximum use of the full dynamic range available to avoid the loss of accuracy caused by discretisation errors.

The camera used in this work was a cooled, monochrome CCD (QImaging QICAM 12-bit) with 1392 × 1040 pixels, 4.65 μm × 4.65 μm pixel size and 1/2" optical format). It has been reported that a minimum bit-depth of 6 bits is adequate for sampling the speckle pattern^4^, which is easily satisfied by our system. The camera was cooled to reduce the dark noise that would otherwise artificially increase the measured speckle contrast. The camera was used with unity gamma correction to ensure a linear relationship between the optical intensity and pixel value. Without this condition, a change in optical intensity would result in an unwanted change in the measured speckle contrast.

Our objective was to design the system to resemble, as closely as possible, the response of the human eye. This is nontrivial as the integration time of the eye differs greatly at distinct positions on the retina. For example, rod cells are very sensitive to low levels of light and consequently integrate over a longer period of time than the less sensitive cones. Moreover, the integration time for the photoreceptor cells decreases with increased illumination intensity and is found to vary considerably across the visible spectrum^34^. Consequently, there is no single value of the camera integration time that would perfectly match human perception. However, for the level of luminance considered in this work (e.g. L = 48 cd/m^2^), photopic vision (cones) dominates over scotopic vision (rods) at the retina and the eyeball moves to ensure that light from the point of primary interest falls on to the region where there is a high density of cone receptors. Furthermore, it has been reported that photopic vision at long wavelengths has a temporal integration time of approximately 50 ms^35^. Therefore, as the eye directs light from its point of interest onto the cone-dense fovea centralis, this integration time was selected unless otherwise stated.

To benchmark our measurements against existing literature the speckle contrast of a highly coherent He–Ne laser without a diffuser was tested and found to be *C* = 0.625 ± 0.008, close to the theoretical value of 1/√2. Another measurement was taken with a RGGD in the diffuser plane tested at 40–200 rpm. It was found that, in this case, *C* = 0.04 ± 0.003, which is in good agreement with previously published results for similar devices^2^. Unless stated otherwise, all values for the speckle contrast are quoted to an accuracy of ± 0.003.

## Supplementary Information

Supplementary Information
