# Supplementary material for: Enhancing laser speckle reduction by decreasing the pitch of a chiral nematic liquid crystal diffuser
Source: Sci Rep. 2021 Mar 1;11:4818. doi: 10.1038/s41598-021-83860-3 (PMC7921095; doi:10.1038/s41598-021-83860-3)
Supplement: Supplementary file 1 — Supplementary Information [file 41598_2021_83860_MOESM1_ESM.docx]

**Supplementary Information**

**Enhancing laser speckle reduction by decreasing the pitch of a chiral nematic liquid crystal diffuser**

David Hansford, Yihan Jin^†^, Steve J. Elston, and Stephen M. Morris*

*Department of Engineering Science, University of Oxford, Parks Road, Oxford, OX3 1PJ, United Kingdom*

*
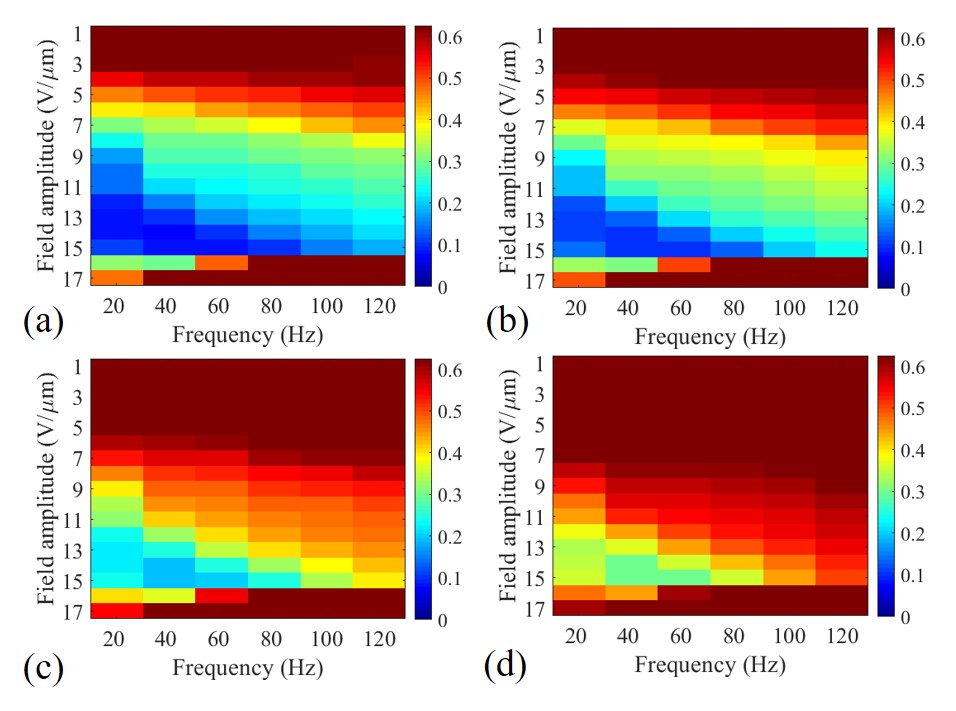
*

**Figure S1:** Speckle contrast maps measured for a chiral nematic LC device recorded for four different CCD camera exposure times: (a) 200 ms, (b) 100 ms, (c) 25 ms, and (d) 10 ms. The cells tested were nominally 20 µm-thick and the cell temperature throughout measurements was T = 25°C.


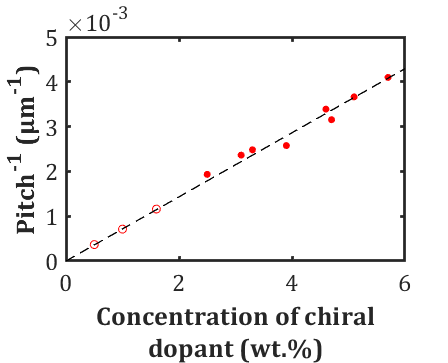


**Figure S2:** Plot of the reciprocal of the pitch (𝜇m^-1^) as a function of the chiral dopant concentration (wt.%) for chiral nematic mixtures consisting of the nematic E7 dispersed with different concentrations of chiral dopant BDH1281. Filled circles represent measured data points. Open circles represent values extrapolated from the best fit.


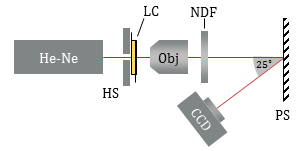


**Figure S3:** A schematic diagram of the experimental setup for measuring the speckle contrast of a laser source after passing through the LC device. (HS) Hot Stage, (LC) Liquid Crystal device, (Obj) Microscope Objective, 10×, 0.3 NA, (NDF) Neutral Density Filter with optical density varied from 0.1 - 2.0, (CCD) Cooled, monochrome, 12-bit camera with 3.2 mm iris and focusing lens (*f* = 35 mm).
